# Ouabain Protects Human Renal Cells against the Cytotoxic Effects of Shiga Toxin Type 2 and Subtilase Cytotoxin

**DOI:** 10.3390/toxins9070226

**Published:** 2017-07-18

**Authors:** María M. Amaral, Magalí C. Girard, Romina S. Álvarez, Adrienne W. Paton, James C. Paton, Horacio A. Repetto, Flavia Sacerdoti, Cristina A. Ibarra

**Affiliations:** 1 Laboratorio de Fisiopatogenia, Departamento de Fisiología, Instituto de Fisiología y Biofísica Bernardo Houssay (IFIBIO Houssay-CONICET), Facultad de Medicina, Universidad de Buenos Aires, Buenos Aires 1121, Argentina; magali.girard@live.com.ar (M.C.G.); roalvarez_4@yahoo.com.ar (R.S.Á.); flasacerdoti@gmail.com (F.S.); cristinaadrianaibarra@gmail.com (C.A.I.); 2 Research Centre for Infectious Diseases, Department of Molecular and Cellular Biology, University of Adelaide, Adelaide 5005, Australia; adrienne.paton@adelaide.edu.au (A.W.P.); james.paton@adelaide.edu.au (J.C.P.); 3 Servicio de Pediatría, Hospital Nacional Profesor Alejandro Posadas, Buenos Aires 1684, Argentina; harepetto@yahoo.com.ar

**Keywords:** shiga toxin, subtilase cytotoxin, Hemolytic Uremic Syndrome, Ouabain, prevention

## Abstract

Hemolytic uremic syndrome (HUS) is one of the most common causes of acute renal failure in children. The majority of cases are associated with Shiga toxin (Stx)-producing *Escherichia coli* (STEC). In Argentina, HUS is endemic and presents the highest incidence rate in the world. STEC strains expressing Stx type 2 (Stx2) are responsible for the most severe cases of this pathology. Subtilase cytotoxin (SubAB) is another STEC virulence factor that may contribute to HUS pathogenesis. To date, neither a licensed vaccine nor effective therapy for HUS is available for humans. Considering that Ouabain (OUA) may prevent the apoptosis process, in this study we evaluated if OUA is able to avoid the damage caused by Stx2 and SubAB on human glomerular endothelial cells (HGEC) and the human proximal tubule epithelial cell (HK-2) line. HGEC and HK-2 were pretreated with OUA and then incubated with the toxins. OUA protected the HGEC viability from Stx2 and SubAB cytotoxic effects, and also prevented the HK-2 viability from Stx2 effects. The protective action of OUA on HGEC and HK-2 was associated with a decrease in apoptosis and an increase in cell proliferation. Our data provide evidence that OUA could be considered as a therapeutic strategy to avoid the renal damage that precedes HUS.

## 1. Introduction

The Hemolytic Uremic Syndrome (HUS) was described in 1973 by Gianantonio et al. as systemic complications characterized by non-immune hemolytic anemia, thrombocytopenia, and acute renal failure (ARF) [1]. Most cases of HUS are associated with a Shiga toxin-producing *Escherichia coli* (STEC) infection called diarrhea-associated HUS [2]. In Argentina, diarrhea-associated HUS is endemic and the incidence rate is the highest in the world, for reasons that are still unknown. In the last 10 years, about 400 cases per year have been registered, with an incidence of 10 to 17 cases per 100,000 children under five years of age, and with a case fatality rate from 1 to 4% [3]. HUS is the most common cause of ARF in children, the second leading cause of chronic renal failure in children younger than five years old, and is responsible for 20% of kidney transplants in children and adolescents [4]. The most significant STEC virulence factors are Shiga toxins (Stx), encoded by genes located in the genome of inducible lambdoid bacteriophages [5]. Stx type 1 and type 2 (Stx1 and Stx2), produced by STEC O157:H7 and non-O157:H7 strains are considered the main virulence factors that cause the renal injury in HUS patients. In particular, STEC strains expressing Stx2 are responsible for the most severe cases of HUS in Argentina [6] and elsewhere.

STEC colonize the intestinal tract of livestock and humans can be infected by STEC through ingestion of undercooked meat, vegetables, or unpasteurized dairy products contaminated with STEC, drinking or swimming in contaminated water, direct contact with animals and person-to-person transmission by the fecal-oral route, all of which are favored by a low infectious dose of STEC (<100 bacteria per gram of food) [7].

Once bacteria are ingested, then colonize the gut and release Stx into the lumen. The toxin can access the systemic circulation and contact the target cells by binding the glycolipid receptor globotriaosylceramide (Gb3) [8]. Then, Stx is internalized into the target cells by endocytosis and the A subunit is cleaved into A1 and A2 fragments in the endoplasmic reticulum (ER). After that, the A1 fragment is translocated into the cytosol where it inactivates the ribosomes leading to protein synthesis inhibition and activation of cell stress response pathways. As a consequence of this event, cell death by apoptosis is triggered [9].

Stx family toxins contain a single 30 kDa A-subunit and a pentamer of noncovalently attached identical 7 kDa B-subunits. While enzymatic activity is located in the A subunit, cell recognition receptor binding properties reside in the B-subunits [10].

Subtilase cytotoxin (SubAB) is another virulence factor released by STEC strains lacking the locus of enterocyte effacement [11,12,13]. It was originally isolated from a serotype O113:H21 STEC strain that was involved in a HUS outbreak in Australia [14] and has also been identified in Argentina [15]. SubAB cytotoxic action depends on the proteolytic cleavage of the ER chaperone BiP (GRP78) [16], which activates a massive ER stress response and finally, promotes apoptosis in eukaryotic cells [17,18,19]. Even though specific host glycoconjugates that act as SubAB receptors are still unknown, this cytotoxin has affinity for glycans terminating in *N*-glycolylneuraminic acid (Neu5Gc) [20], so this monosaccharide is considered an essential component of SubAB receptors. Although humans cannot synthesize Neu5Gc, they can incorporate it through dietary intake. These glycans, also have been found in Vero [21] and HeLa cells [22].

The importance of SubAB in the physiopathology of STEC disease is still unknown, but intraperitoneal injection of the purified toxin induces the salient features of HUS in a murine model [23]. Even though SubAB has not been detected in the blood of patients, numerous STEC serotypes expressing SubAB have been associated with severe cases of HUS around the world [15].

In previous studies, our group has shown that, in vitro, exposure to Stx2 or SubAB decreases viability of human renal cells such as human glomerular endothelial cells (HGEC) and immortalized human proximal tubule epithelial cells (HK-2) and this was associated with the induction of apoptosis [24,25,26]. Also, both toxins inhibited water absorption across HGEC and HK-2 monolayers [27]. Stx2 has been reported to cause apoptosis by increasing the expression of the pro-apoptotic factor Bax [28], while Paton et al. also demonstrated that SubAB triggers apoptosis due to the induction of the pathway dependent on the pro-apoptotic Bax/Bak proteins [18].

Aperia et al. proposed that the interaction between the cardiotonic steroid ouabain (OUA) [29] and Na/K ATPase triggers an intracellular signaling mechanism, which may interfere with apoptotic processes [30]. OUA is reported to bind the α subunit of Na/K ATPase, so it contacts the inositol 1,4,5-tri-phosphate receptor (IP3R) and finally triggers slow intracellular calcium oscillations [31,32,33]. These signals developed by OUA induce a down-regulation of the apoptotic factor Bax and an up-regulation of the anti-apoptotic factor Bcl-xL. Nanomolar (nM) concentrations of OUA are able to upset the balance between Bax and Bcl-xL and, as a consequence; this event prevents rat renal epithelial cells from undergoing apoptosis caused by Stx2. In addition, in vivo experiments demonstrate that treatment with OUA protects mice inoculated with Stx2 from the renal damage [34].

It is well known that kidneys are the most affected organs in diarrhea-associated HUS [24,35] and, up to now, neither licensed vaccine nor effective therapy for diarrhea-associated HUS has been available for humans. Considering this serious problem, in this study, we investigated whether treatment with nM concentrations of OUA could be an alternative therapy to prevent the apoptosis caused by Stx2 and SubAB in order to avoid renal damage.

## 2. Results

### 2.1. Establishment of OUA Non-Cytotoxic Concentrations for Renal Cells

**Figure 1:**
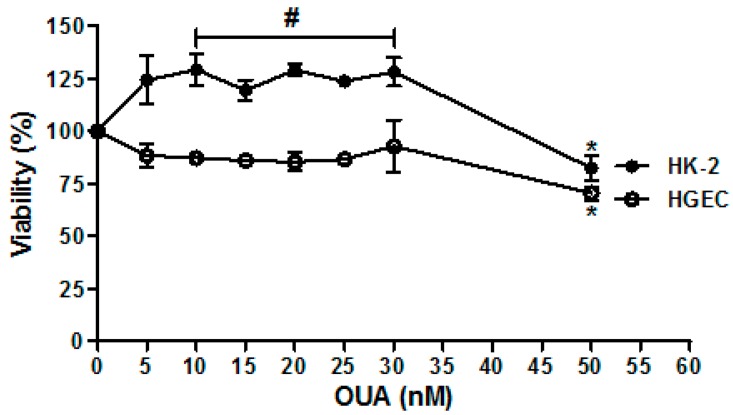
Oubain (OUA) non-cytotoxic concentrations. Human glomerular endothelial cells (HGEC) and human proximal tubule epithelial cells (HK-2) placed in 96-well plates were exposed to between 5 nM and 50 nM OUA for 72 h. Then, cells were incubated with neutral red for an additional 1 h at 37 °C in 5% CO_2_. Absorbance of each well was read at 540 nm. One hundred percent represents cells incubated under identical conditions but without OUA treatment (Ctrl). Results are expressed as means ± SEM of five experiments, OUA concentrations >30 nM vs. Ctrl, * *p* < 0.05; HK-2: OUA (10–30 nM) vs. Ctrl, # *p* < 0.05.

First, we established non-cytotoxic concentrations of OUA for renal cells by developing cell viability assays. Non-cytotoxic concentrations ranged from 5 to 30 nM as cell viability of HGEC and HK-2 was not affected. Concentrations of OUA above 30 nM were cytotoxic for both HGEC and HK-2 cells (Figure 1) (*n* = 5, * *p* < 0.05). In addition, OUA caused a significant increase in the HK-2 viability (25 %) in a range from 10 to 30 nM (*n* = 5, * *p* < 0.05) (Figure 1).

### 2.2. OUA Protected Human Renal Cell Viability from Cytotoxic Effects of Stx2 and SubAB

**Figure 2:**
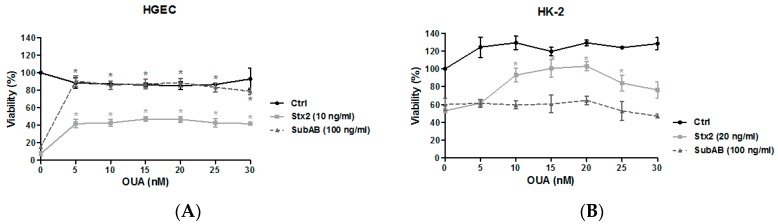
OUA protection on HGEC and HK-2 exposed to toxins. HGEC (**A**) and HK-2 (**B**) placed in 96-well plates were pre-treated with between 5 nM and 30 nM OUA for 24 h, and then incubated with Stx type 2 (Stx2) (Stx2 (10 ng/mL for HGEC or 20 ng/mL for HK-2) or subtilase cytotoxin (SubAB) (100 ng/mL) in the presence of OUA for other 48 h. Then, cells were incubated with neutral red for an additional 1 h at 37 °C in 5% CO_2_. Absorbance of each well was read at 540 nm. One hundred percent represents cells incubated under identical conditions but without OUA or toxins treatment (Ctrl). Results are expressed as means ± SEM of five experiments, OUA + Stx2 vs. Stx2 or OUA + SubAB vs. SubAB, * *p* < 0.05.

We then analyzed the protective effect of OUA on HGEC and HK-2 exposed to Stx2 or SubAB by cell viability assays. Cells were preincubated with OUA (5–30 nM) for 24 h and then incubated with either Stx2 (10 ng/mL for HGEC and 20 ng/mL for HK-2) or SubAB (100 ng/mL) in the presence of OUA for an additional 48 h. The inhibition of HGEC viability caused by Stx2 or SubAB was significantly lower when the cells were treated with OUA (Figure 2A). The maximum prevention of cytotoxicity was approximately 50% for Stx2 and 90% for SubAB (Table 1). In addition, OUA also protected HK-2 viability from Stx2 but it did not prevent the SubAB effects (Figure 2B and Table 2). The highest protection from Stx2 was 100%. Furthermore, for both types of cells the maximum protection from the effects of the toxins was obtained with 20 nM OUA (*n* = 5, * *p* < 0.05).

### 2.3. OUA Prevented Morphologic Alterations and Cell Detachment Induced by Stx2 and SubAB 

**Figure 3:**
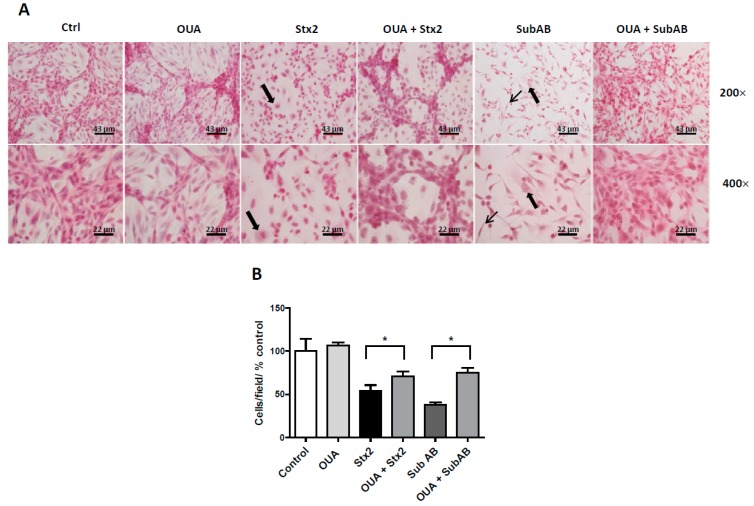
HGEC are protected from toxins morphologic disturbances. HGEC seeded on gelatin-coated glass coverslips were pre-treated with 20 nM OUA for 24 h and then incubated with 10 ng/mL Stx2 or 100 ng/mL SubAB in the presence of OUA for another 48 h. Finally, cells were stained with H&E and the morphology (**A**) and number of HGEC (**B**) were analyzed by light microscopy (×200 and ×400). Results are expressed as means ± SEM of three experiments. One hundred percent represents values of controls. OUA vs. Ctrl ns, OUA + Stx2 vs. Stx2 or OUA + SubAB vs. SubAB, * *p* < 0.05. Thick arrows: intracellular edema. Thin arrows: elongated shape.

**Figure 4:**
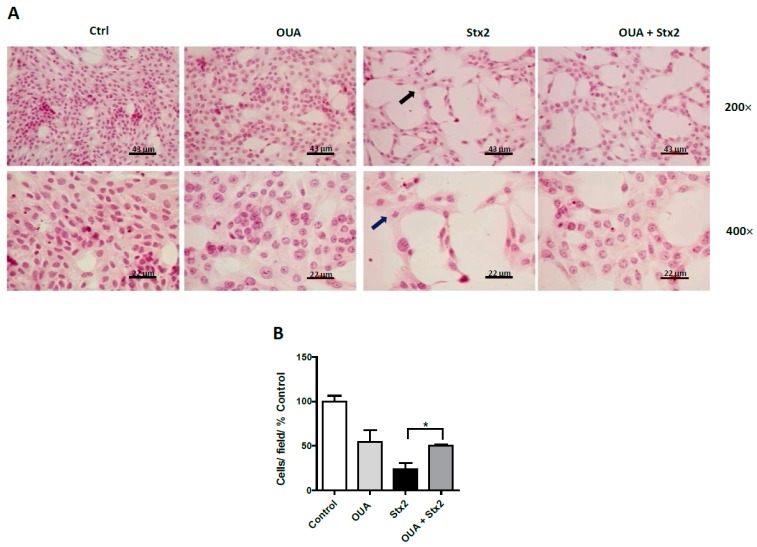
HK-2 are protected from Stx2 morphologic disturbances. HK-2 seeded on glass coverslips were pre-treated with 20 nM OUA for 24 h and then incubated with 20 ng/mL Stx2 and in the presence of OUA for an additional 48 h. Finally, cells were stained with H&E and the morphology (**A**) and number of HK-2 (**B**) were analyzed by light microscopy (×200 and ×400). Results are expressed as means ± SEM of three experiments. One hundred percent represents values of cells control. OUA vs. Ctrl ns, OUA + Stx2 vs. Stx2, * *p* < 0.05. Thick arrows: intracellular edema.

Subsequently, we analyzed the morphology of HGEC and HK-2 treated with OUA (20 nM) and incubated with the toxins. Figure 3A shows that the morphological alterations induced by Stx2 and SubAB in HGEC, such as intracellular edema and elongated shape, were avoided with OUA treatment. Furthermore, OUA prevented about 32% and 54% of the detachment of HGEC caused by Stx2 and SubAB, respectively (Figure 3A,B). Finally, OUA also prevented about 87% of the detachment and the morphological alterations of HK-2 cells caused by Stx2 (Figure 4A,B).

### 2.4. OUA Did Not Affect Na/K-ATPase Function

**Figure 5:**
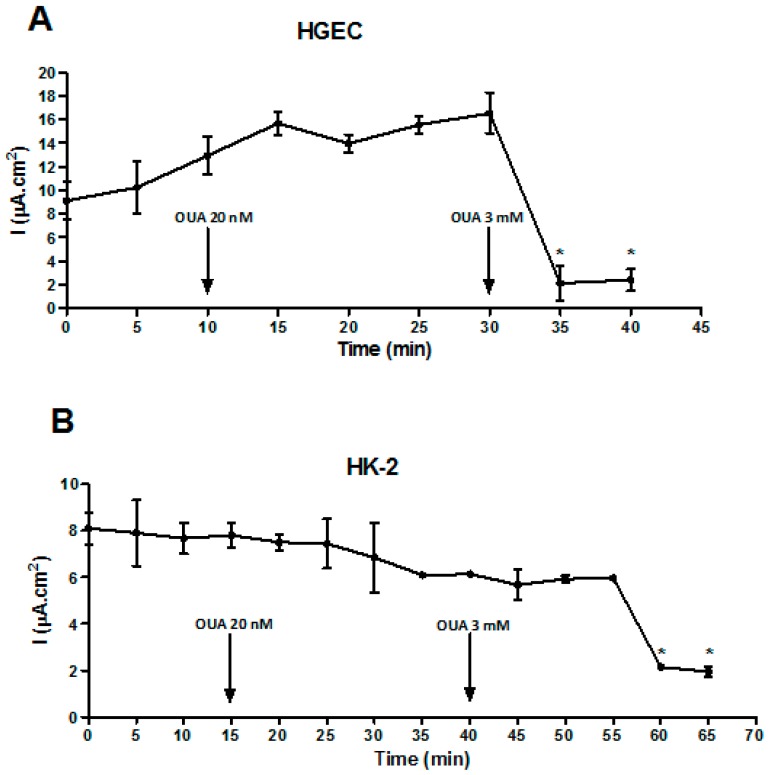
OUA (20 nM) did not modify the electric current across HGEC and HK-2. HGEC (**A**) and HK-2 (**B**) were grown to confluence on Millicell cell culture inserts. Then, TEER and V were measured and values were recorded every 5 min. Following this, the current (I = mA·cm^2^) was calculated before and after treatment with OUA at 20 nM or 3 mM. Results are expressed as means ± SEM of three experiments. 3 mM OUA vs. 20 nM OUA, * *p* < 0.05.

Taking into account that OUA concentrations above the physiological range (nanomolar-picomolar) could affect Na/K-ATPase pump functionality [36], we evaluated the pump function by measuring the electrical current across HGEC and HK-2 cell monolayers (Figure 5). Electrical current values were not modified before and after the addition of 20 nM OUA (HGEC: 12.9 ± 1.6 µA/cm^2^ vs. 15.5 ± 0.75 µA/cm^2^; HK-2: 7.8 ± 0.5 µA/cm^2^ vs. 6.8 ± 1.5 µA/cm^2^, NS *n* = 3) while 3 mM OUA caused a significant decrease of this parameter (HGEC: 16.5 ± 1.7 µA/cm^2^ vs. 2.4 ± 0.9 µA/cm^2^; HK-2: 6.1 ± 0.1 µA/cm^2^ vs. 1.9 ± 0.2 µA/cm^2^, *p* < 0.01, *n* = 3).

### 2.5. OUA Prevented Apoptosis and Necrosis Caused by Stx2 and SubAB

**Figure 6:**
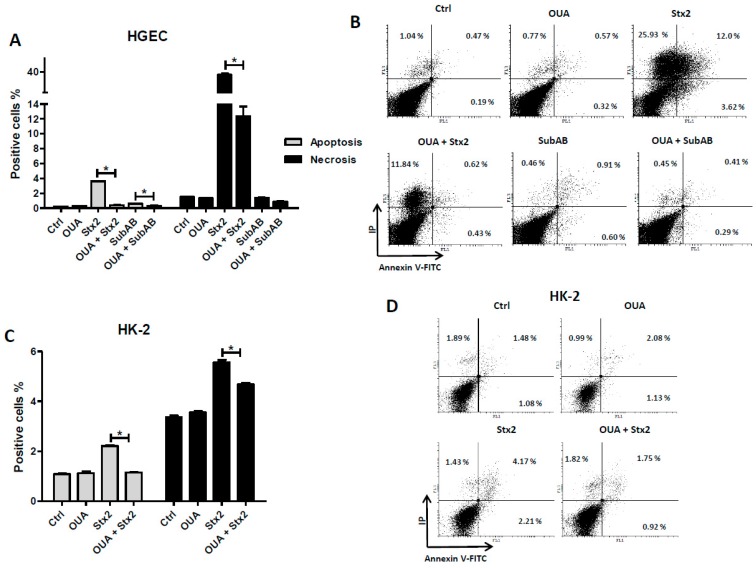
Prevention of apoptosis and necrosis by OUA. Annexin V-FITC/IP double staining assay was used to quantify necrosis and apoptosis by flow cytometry. HGEC (**A**) and HK-2 (**B**) cells were pre-treated with 20 nM OUA for 24 h and then incubated with Stx2 (10 ng/mL for HGEC or 20 ng/mL for HK-2) or SubAB (100 ng/mL for HGEC) and in the presence of OUA for an additional 48 h. Then, cells were labeled with Annexin V-FITC/IP for 10 min. Results are expressed as means ± SEM of three experiments. OUA vs. Ctrl ns, OUA + Stx2 vs. Stx2 or OUA + SubAB vs. SubAB, * *p* < 0.05. A representative experiment is shown in panels (**C**,**D**).

In order to study the possible mechanism(s) triggered by OUA involved in the protection of renal cells from Stx2 and SubAB cytotoxicity, we determined apoptotic activity in HGEC and HK-2 cells treated with Stx2 or SubAB in the presence of OUA. Cells were treated with OUA and toxins following the protocol described above, and were then labelled with AV and IP and analyzed by flow cytometry. Treatment of HGEC and HK-2 with 20 nM OUA had no have effects per se on apoptosis and necrosis (Figure 6). OUA significantly decreased the percentage of apoptotic and necrotic HGEC after OUA + Stx2 treatment compared to Stx2 (Figure 6A,B). In addition, OUA also protected HGEC from apoptosis but not from necrosis caused by SubAB (Figure 6A,B). Furthermore, OUA treatment prevented the apoptosis and necrosis of HK-2 cells caused by Stx2 (Figure 6C,D).

### 2.6. Cell Proliferation Was Stimulated by OUA

**Figure 7:**
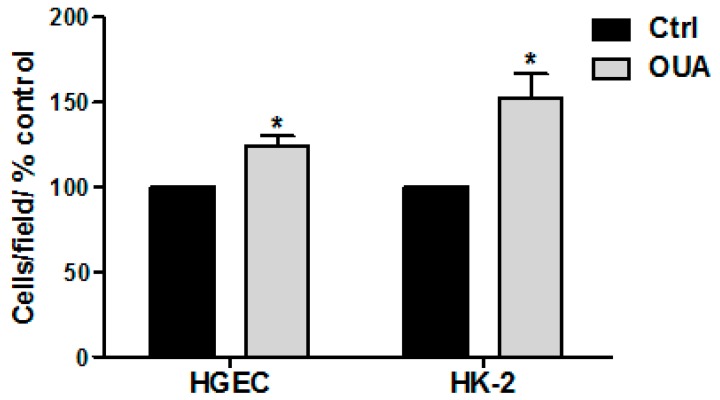
OUA increases the number of viable HGEC and HK-2. HGEC and HK-2 were grown to confluence in 6-well plates and then treated with OUA (20 nM) for 72 h. After that, cells were trypsinized, centrifuged and resuspended with 0.4% trypan blue. Finally, cells were counted into a Neubauer chamber. Results are expressed means ± SEM of three experiments. OUA vs. Ctrl * *p* < 0.05.

Since it was proposed that nM OUA concentrations stimulate cell proliferation and increase cell viability in different types of cells [32], we examined whether the protection of OUA over human renal cells incubated with the toxins could be associated with the stimulation of cell proliferation. Thus, proliferation studies on HGEC and HK-2 treated with 20 nM OUA were developed first by cell count with Trypan Blue. Figure 7 shows that OUA caused a significant increase in the number of viable cells relative to the control, indicating that OUA is promoting HGEC and HK-2 proliferation.

**Figure 8:**
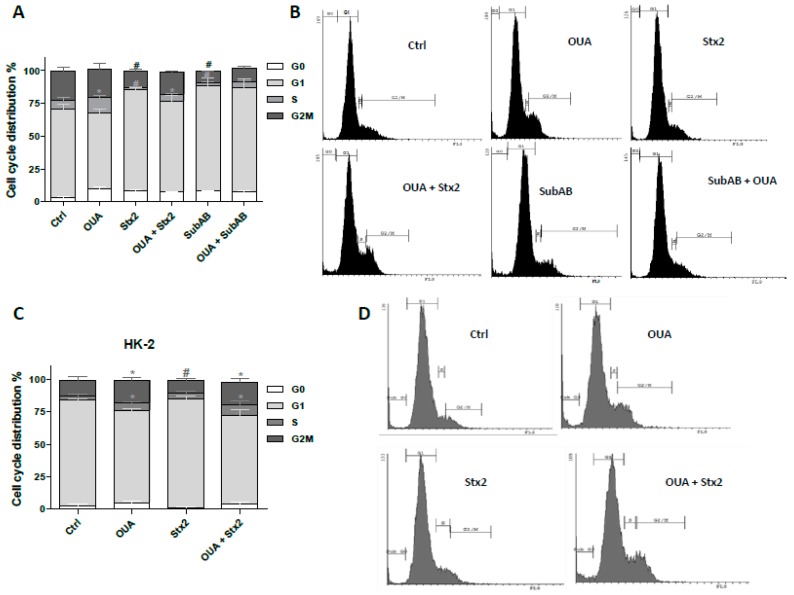
OUA stimulated HGEC and HK-2 proliferation. HGEC (**A**) and HK-2 (**C**) were pre-treated with 20 nM OUA for 24 h. Then, cells were incubated with 10 ng/mL Stx2 or 100 ng/mL SubAB (for HGEC) or 20 ng/mL Stx2 (for HK-2), and in the presence of OUA for an additional 48 h. After treatment, cells were fixed with 70% ethanol, incubated with PI (50 μg/mL) for 30 min and then analyzed by flow cytometry. The cell cycle phases were analyzed by Cyflogic software. Results are expressed as means ± SEM of three experiments. OUA vs. Ctrl or OUA + Stx2 vs. Stx2, * *p* < 0.05. OUA + SubAB vs. SubAB, ns. Stx2 vs. Ctrl or SubAB vs. Ctrl, # *p* < 0.05. A representative experiment is shown in panels (**B**,**D**).

To confirm this result, the effects of OUA on the cell cycle phases of HGEC and HK-2 cells were analyzed by IP-labeling and flow cytometry. As shown in Figure 8A,B, both toxins caused a significant decrease in the percentage of HGEC in the S phase (Stx2: 1.8 ± 0.2% and SubAB: 2.7 ± 0.4% vs. Ctrl: 5.3 ± 2.4%) and in HGEC proliferation, G2M phase (Stx2: 12.0 ± 1.5 % and SubAB: 8.3 ± 0.5% vs. Ctrl: 21.4 ± 3.2%). On the other hand, OUA increased the percentage of HGEC in the S phase with respect to Ctrl, and it was able to prevent the effects of Stx2, as shown for the G2M phase in OUA + Stx2 treatment compared to Stx2 (17.3 ± 0.9% vs. 12.0 ± 1.5 %). Figure 8C,D shows that Stx2 also decreased the percentage of HK-2 in G2M phase (Stx2: 9.6 ± 1.5% vs. Ctrl: 12.3 ± 3.0%) and OUA prevented this effect (Stx2 + OUA: 16.7 ± 3.1% vs. Stx2: 9.6 ± 1.5%). In addition, OUA per se induced an important increase in proliferating HK-2 (HK-2: 17.6 ± 2.1% vs. Ctrl: 12.3 ± 3.0%).

## 3. Discussion

Diarrhea-associated HUS is an infectious disease, most frequently caused by STEC infection. The principal organ affected is the kidney, which is a consequence of the action of Stx2 on endothelial and epithelial cells that triggers cell death by apoptosis. To date, this pathology cannot be easily avoided, and nor is an effective vaccine or treatment available. In this context, it has been postulated that OUA signaling may interfere with the toxin-mediated apoptosis by down-regulation of the apoptotic factor Bax and up-regulation of the anti-apoptotic factor Bcl-xL [30,31,32,33,34,37]. Thus, the aim of this work was to evaluate if OUA at nM concentrations is able to prevent the renal damage caused by Stx2 and SubAB.

We used primary human renal endothelial cells (HGEC) and the epithelial cell line HK-2 in this study because we have previously characterized these cells as highly sensitive to cytotoxic effects caused by Stx2 and SubAB [24,25,27]. We have found that 20 nM of OUA was non-cytotoxic for both cell types and that it is able to prevent the loss of viability of HGEC and HK-2 cells due to Stx2 cytotoxicity. Furthermore, 20 nM OUA also reduced the morphological alterations and detachment induced by this toxin in both cell types. We also demonstrated that the electrical current through HGEC and HK-2 monolayers was not altered by 20 nM OUA. These results suggest that 20 nM OUA is able to protect HGEC and HK-2 from the cytotoxic effects of Stx2 without altering the function of Na/K-ATPase pump. 

On the other hand, although SubAB cytotoxicity to HGEC was also prevented by OUA, these effects could not be prevented in HK-2 cells. It is possible that different features of HK-2 could influence the response of these cells to OUA. For example, Na/K-ATPase activity and tyrosine phosphorylation were stimulated by OUA in HKC-5 and HKC-11, but not in HK-2 cells [38]. Alternatively, the ability of OUA to prevent the Stx2-mediated, but not the SubAB-mediated, cytotoxicity on HK-2 cells could be related to differences in the mechanisms used by the two toxins to activate apoptosis [19,39]. Results from the current study also demonstrated that OUA caused a marked decrease in the number of apoptotic HGEC and HK-2 cells, in accordance with previous results obtained by Burlaka et al. [34] for rat renal epithelial cells, which showed that nM concentrations of OUA protected these cells from Stx2-triggered apoptosis.

The current study also determined that OUA was able to increase the number of viable HGEC and HK-2 cells, although this increase was more important in HK-2 than HGEC. In addition, 20 nM OUA increased the percentage of proliferating HK-2 cells. These observations were consistent with other reports showing the proliferation of kidney cells from rat and monkey in the presence of OUA [32]. Regarding to the molecular events involved in the OUA-mediated cell proliferation, it has been shown that OUA stimulates Na-K-ATPase-mediated ^86^Rb uptake and cell proliferation through the activation of a signaling cascade involving Src kinase, ERK, and Akt in a cell culture model of proximal tubule cells (OK cells) [40,41].

In addition, OUA was able to prevent the effect of Stx2 on the HGEC and HK-2 cell cycle, and also the effect of SubAB on the HGEC cell cycle, by stimulating cell proliferation, suggesting that OUA protection is a consequence not only on cellular apoptosis but also on proliferation.

In summary, our results demonstrated that nM concentrations of OUA are able to protect the viability of HGEC and HK-2 from Stx2 cytotoxicity. In addition, it protects HGEC viability from SubAB, but is not effective against this toxin in HK-2 cells. These protective effects can be attributed to the prevention of apoptosis and, additionally, as a consequence of an increase in cell proliferation. Further experiments in animal models of HUS are in progress to evaluate whether OUA is able to prevent the cytotoxic effects of Stx2 and SubAB on the kidney in vivo.

## 4. Conclusions

Considering that the average interval between ingestion of STEC and onset of HUS manifestations is approximately 3–5 days [42], this period of time could be taken as a window of opportunity for potential therapeutic use of nM concentrations of OUA to avoid the renal injury that precedes HUS.

## 5. Materials and Methods

### 5.1. Reagents

Purified Stx2a was provided by Phoenix Laboratory, Tufts Medical Center, Boston, MA, USA. SubAB was purified from recombinant *E. coli* by Ni-NTA chromatography (ProBond, Invitrogen, Carlsbad, CA, USA) via a His_6_ tag fused to the C-terminus of the B subunit, as described previously [14]. Purity was greater than 98%, as judged by SDS-PAGE and staining with Coomassie Blue. Ouabain (C_29_H_44_O_12_) was obtained from MP Biomedicals, Solon, OH, USA.

### 5.2. Primary Culture

Human glomerular endothelial cells (HGEC) were isolated from kidney fragments removed from normal areas from different pediatric patients with segmental uropathies, or a tumor in one pole and normal creatinine, that were undergoing nephrectomies performed at Hospital Nacional “Alejandro Posadas”, Buenos Aires, Argentina. Written informed consent was obtained from the next of kin, caretakers, or guardians on the behalf of the minors/children participating in our study. The study was conducted in accordance with the Declaration of Helsinki, and the protocol was approved by the Ethics Committee “Dr. Vicente Federico Del Giúdice” of the Hospital Nacional “Alejandro Posadas”. Endothelial cells were isolated as was previously described [24]. For growth-arrested conditions, a medium with a half of the fetal calf serum (FCS) concentration (10%) and without endothelial cell growth supplement (ECGS) was used. For the experiments, cells were used between 2–7 passages, after characterization for von Willebrand factor (VWF, DAKO, Tecnolab S.A., Buenos Aires, Argentina) and platelet/endothelial cell adhesion molecule 1 (PECAM-1, DAKO, Tecnolab S. A., Buenos Aires, Argentina) positive expression [24].

### 5.3. Cell Lines Culture

Human proximal tubular epithelial cell line (HK-2) was grown in DMEM/F12 medium (Sigma Aldrich, St. Louis, MO, USA) containing 10% FCS, 100 U/mL penicillin/streptomycin (GIBCO, Invitrogen, Carlsbad, CA, USA), 2 mM L-glutamine, 15 mM HEPES at 37 °C in a humidified 5% CO_2_ incubator. A medium without FCS was used in growth-arrested conditions.

All the experiments were performed at growth-arrested conditions.

### 5.4. Neutral Red Cytotoxicity Assay

The neutral red cytotoxicity assay was adapted from previously described protocols [43]. Vero, HGEC and HK-2 cells were placed in 96-well plates and grown to confluence in complete medium. 

To establish the OUA non-cytotoxic range of concentrations, HGEC and HK-2 cells were treated with OUA (from 5 to 50 nM) every 24 h during 72 h. To evaluate the effect of OUA on Stx2 and SubAB cytotoxicity, HGEC and HK-2 were pretreated with OUA for 24 h and then cells were incubated with or without Stx2 (10 ng/mL for HGEC or 20 ng/mL for HK-2) or SubAB (100 ng/mL) and in the presence of OUA during an additional 48 h. After treatments, freshly diluted neutral red (Sigma Aldrich, St. Louis, MO, USA) was added to a final concentration of 10 mg/mL and then cells were incubated for an additional 1 h at 37 °C in 5% CO_2_. Cells were then washed and fixed with 200 mL of 1% CaCl_2_/1% formaldehyde and then lysed with 200 mL 1% acetic acid in 50% ethanol whereby neutral red was solubilized. Absorbance in each well was measure in an automated plate spectrophotometer at 540 nM. Results were expressed as percentage of viability, where 100% represents cells incubated under identical conditions but without OUA or toxin treatment. For experiments studying the action of OUA on cells incubated with Stx2 or SubAB, results were expressed as percentage of cell death prevention, where 0% represents cells incubated under identical conditions but without OUA treatment.

### 5.5. Cell Morphology Analysis

HGEC and HK-2 cells were seeded on glass coverslips (12 mm), washed with PBS at pH 7.4 and pre-treated with OUA (20 nM) as previously in growth-arrested conditions for 24 h. Cells were then exposed to Stx2 (10 ng/mL for HGEC or 20 ng/mL for HK-2) or SubAB (100 ng/mL for HGEC) in the presence of OUA for 48 h. Finally, cells were fixed for 2 h at room temperature with 96% *v*/*v* alcohol, stained with Hematoxylin/Eosin (H&E) and observed by light microscopy (×200 and ×400, Zeiss Axiophot, Zeiss, Heidelberg, Germany). Cell counts were performed on 10 fields at ×200 magnification. Results are expressed as means ± SEM of three experiments. One hundred percent represents values of control cells.

### 5.6. Na/K-ATPase Activity

HGEC and HK-2 were grown to confluence on a permeable support (Millicell cell culture inserts (PIHP01250, Millipore, Merck KGaA, Darmstadt, Germany). Then, the electrical resistance (TEER) and the voltage (V) across the endothelial and epithelial monolayers were measured with a Millicell-ERS electric resistance system (Millipore, Billerica, MA, USA) calibrated for each measurement. TEER and V values were recorded every 5 min. TEER is expressed as Ω·cm^2^ (filter area: 1.13 cm^2^). The current (I) was calculated before and after OUA treatment (20 nM OUA or 3 mM [44]), according with the Ohm’s law, I = V/R.

### 5.7. Necrosis and Apoptosis

HGEC and HK-2 cells (5 × 10^4^ cells) were seeded in 6-well plates and grown to confluence. Then, cells were pre-treated with OUA (20 nM) as previously. After that, cells were exposed to Stx2 (10 ng/mL for HGEC or 20 ng/mL for HK-2) or SubAB (100 ng/mL for HGEC) in the presence of OUA for 48 h. Subsequently, detached cells were collected from supernatant and attached cells were trypsinized. Finally total cells from each well were washed with PBS at pH 7.4. The percentage of necrotic and apoptotic cells was established by Annexin V-FITC/PI double staining assay and flow cytometry analysis. For that, cells were resuspended in binding buffer (0.1 M Hepes, 0.14 M NaCl, 25 mM CaCl_2_) and FITC-conjugated annexin V and PI (propidium iodide) were added. The mixture was incubated for 10 min at room temperature, cells (approximately 50,000 events) were acquired by a Partec model PAS III flow cytometer and data were analyzed by Cyflogic software (version 1.2.1., CyFlo Ltd PL 634 20701, Turku, Finland). The results were interpreted as follows: negative cells for both PI and Annexin-V-FITC staining were considered live cells; PI negative/Annexin-V-FITC-positive stained cells were considered in early apoptosis; PI-positive/Annexin-V-FITC positive or PI-positive/Annexin V-negative-stained cells were considered in necrosis.

### 5.8. Cell Proliferation Studies

#### 5.8.1. Trypan Blue Assay

The number of viable cells was estimated by Blue Trypan dye that identifies nonviable cells by penetrating their damaged cell membrane. For that, HGEC and HK-2 cells (5 × 10^4^ cells) were placed in 6-well plates, grown to confluence in complete medium and then treated with OUA (20 nM) for 72 h. After that, cells were trypsinized, centrifuged and resuspended. An aliquot of 10 μl of each sample was then mixed with 90 μL of 0.4% trypan blue solution (Sigma–Aldrich, St. Louis, MO, USA) and incubated for 2 min at room temperature. The cell suspension was loaded into a Neubauer chamber and cells were counted. Results are expressed as the percentage of the number of cells in each treatment respect to the percentage of number of control cells.

#### 5.8.2. Cell Cycle

HGEC and HK-2 (5 × 10^4^ cells) cells were placed in 6-well plates and grown to confluence in complete medium. Cells were pre-treated with OUA (20 nM) as previously in growth-arrested conditions for 24 h. Then cells were exposed to Stx2 (10 ng/mL for HGEC or 20 ng/mL for HK-2) or SubAB (100 ng/mL for HGEC) in the presence of OUA for 48 h. After treatment, cells were trypsinized, washed twice with ice-cold PBS, fixed with 70% ethanol at −20 °C overnight, washed again and then incubated with PI (50 μg/mL) for 30 min at room temperature in the dark. The cells were resuspended in 500 μL PBS and then acquired by a flow cytometer. The cell cycle phases were analyzed by Cyflogic software. Results are expressed as positive cells % for each cycle phase: sub G0 arrested cells or outside of the cell cycle; G1: inside the cell cycle; S: replicating cells or DNA synthesis; G2/M: proliferating cells.

### 5.9. Data Analysis

Data are presented as mean ± SEM. Statistical analysis was performed using the Graph Pad Prism Software 5.0 (San Diego, CA, USA). One-way ANOVA was used to calculate differences between groups and Tukey’s multiple comparisons test was used as an a posteriori test. For cell cycle experiments two-way ANOVA was used to calculate differences between groups and Bonferroni post tests were used as an a posteriori test. Statistical significance was set at *p* < 0.05.

## Figures and Tables

**Table 1:** Stx2 and SubAB cytotoxic effects on HGEC treated with OUA. * *p* < 0.05, *n* = 5 (Stx2 + OUA vs. Stx2; SubAB + OUA vs. SubAB).

Treatment	Cytotoxicity
Stx2 (10 ng/mL)	92.6 ± 2.3
SubAB (100 ng/mL)	84.9 ± 1.8
Stx2 + OUA	53.0 ± 3.3 *
SubAB + OUA	11.4 ± 4.8 *

**Table 2:** Stx2 and SubAB cytotoxic effects on HK-2 treated with OUA. * *p* < 0.05, *n* = 5 (Stx2 + OUA vs. Stx2; SubAB + OUA vs. SubAB).

Treatment	Cytotoxicity
Stx2 (20 ng/mL)	47.0 ± 2.2
SubAB (100 ng/mL)	40.0 ± 7.0
Stx2 + OUA	0 *
SubAB + OUA	35.3 ± 4.6 *
